# A humanisation approach for the management of Joint Hypermobility Syndrome/Ehlers-Danlos Syndrome-Hypermobility Type (JHS/EDS-HT)

**DOI:** 10.1080/17482631.2017.1371993

**Published:** 2017-09-03

**Authors:** Carol J. Clark, Isobel Knight

**Affiliations:** ^a^ Department Human Sciences and Public Health, Faculty of Health and Social Sciences, Bournemouth University, Bournemouth, UK; ^b^ Private Bowen Practitioner, Hamilton House, London, UK

**Keywords:** Hypermobility, well-being, humanisation framework, condition management, chronic pain

## Abstract

Joint Hypermobility Syndrome/Ehlers-Danlos Syndrome—Hypermobility Type (JHS/EDS-HT) is a complex and multisystemic condition which significantly impacts on a person’s health and well-being and is challenging for health professionals (HPs) to manage. People with JHS/EDS-HT and HPs recognise the individual nature and the complexities of the condition. There is a requirement to understand the condition within the context of the individual human dimensions of illness and healing. The aim of this paper is to explore the management of this condition using a theoretical model referred to as the Humanisation Framework.  It is suggested that using the philosophical dimensions of this framework will empower HPs and those with JHS/EDS-HT to work together to proactively manage this condition. The eight dimensions of the Humanisation Framework facilitate an experiential understanding of the person within their context and environment, providing a constructive adjunct to the evidence-based management of those with JHS/EDS-HT. The humanisation framework was developed for health and social care and uses the philosophy behind well-being and what makes well-being possible. This paper explores how HPs may use aspects of the framework to understand the condition and empower and motivate those with JHS/EDS-HT to be active participants in their own well-being.

## Introduction

Joint Hypermobility Syndrome/Ehlers-Danlos Syndrome-Hypermobility Type (JHS/EDS-HT) is a multisystemic condition commonly seen by health professionals (HPs) (Clark & Simmonds, 2011; Connelly, Hakim, Davenport, & Simmonds, 2015). Those with the condition report generalized joint hypermobility and symptoms like musculoskeletal complaints, recurrent joint dislocations/joint instability and chronic pain, sometimes starting in childhood (Adib, Davies, Grahame, Woo, & Murray, 2005; Clinch & Ecclestone, 2009; Malfait et al., 2017). Physiotherapy is considered to be the main mode of treatment (Simmonds & Keer, 2007), although there is little evidence relating to the success of these interventions (Palmer, Bailey, Barker, Barney, & Elliott, 2014); the reason for this is likely to be the complex individual nature of the condition. Those with JHS/EDS-HT feel they would benefit from consulting with HPs who employ a holistic approach and are open and non-judgmental about a person’s story (Knight, 2015; Palmer et al., 2016).

The aim of this paper is to explore the use of the humanisation framework proposed by Todres, Galvin and Holloway (2009) to guide HPs in exploring a person’s story and facilitating the management of JHS/EDS-HT. Todres et al. (2009) describe a framework for humanising care by providing eight philosophically informed dimensions that could be used to guide the HPs in their consultation. This framework should be used as an adjunct to evidence-based consultations and interventions thereby increasing the effectiveness of healthcare.

## Joint Hypermobility Syndrome/Ehlers-Danlos Syndrome-Hypermobility Type (JHS/EDS-HT)

Joint Hypermobility Syndrome/Ehlers-Danlos Syndrome-Hypermobility Type is one of the heritable connective tissue disorders showing symptom overlap with Marfans Syndrome, Osteogeneis Imperfecta, Chronic Fatigue Syndrome and Fibromyalgia (Hakim, Malfait, De Paepe, & Sahota, 2010). Joint Hypermobility Syndrome is considered to be synonymous with EDS-hypermobility type (Grahame, 2013; Tinkle et al., 2009). An updated classification of the diagnostic criteria for the various Ehlers-Danlos syndromes has been published (Malfait et al., 2017), which includes hypermobile Ehlers-Danlos syndrome. In the medical literature it might also be referred to as Hypermobility Syndrome, but, for consistency, in this paper it will be referred to as JHS/EDS-HT.

The musculoskeletal features and chronic pain are well acknowledged in the Villefranche revised nosology and Brighton diagnostic criteria (Beighton, De Pape, Steinmann, Tsipouras, & Wenstrup, 1998; Grahame, Bird, & Child, 2000). More recently the breadth of symptoms have been found to include: Postural Orthostatic Tachycardic Syndrome, gastrointestinal disorders, fatigue and Development Coordination Disorder (Castori, Morlino, Pascolini, Blundo, & Grammatico, 2015; Clark, Khattab, & Carr, 2014; Farmer & Aziz, 2010). The clinical presentation of JHS/EDS-HT is complex, often impacting on a person’s daily activity, family life, social and employment opportunities. There is often a long history of engagement with a variety of HPs. Those encounters with HPs may not always be viewed positively as those with JHS/EDS-HT have reported feeling disbelieved and discredited, which has led to feelings of frustration and dissatisfaction with HPs (Knight, 2015; Palmer et al., 2016). Health professionals find diagnosing JHS/EDS-HT challenging as they may be unaware of the condition or sceptical that the condition exits (Lyell, Simmonds, & Deane, 2016).

Those with JHS/EDS-HT have felt that HPs struggle to manage the condition as it does not fit with their “model of acute injury and recovery” and HPs report there is no healthcare pathway for the management of JHS/EDS-HT (Palmer et al., 2016). Health professionals may have difficulties coming to terms with the individual complexity of the condition and feel overwhelmed by the person’s story. Listening to a person’s story is integral to the management of long-term conditions and it is suggested there is a requirement to understand this within the context of “human dimensions of illness and healing” (Knight, 2015; Nielsen, 2014; Nowaczyk, 2012). In this paper we aim to introduce the reader to the concept of managing those with JHS/EDS-HT using the philosophical concepts of the humanisation framework (Todres et al., 2009).

## The humanisation framework

The framework considers eight dimensions relating to a complex existential phenomenon about what it is to be human (see Table 1). This framework was developed for health and social care (Todres et al., 2009) and considers what makes well-being possible. Understanding that well-being is more than just an absence of illness that it is associated with both an “experience of” and “feeling of” “being-in- the- world” (Galvin & Todres, 2013). It is suggested that considering this framework in practice enables the human elements of care to have a “voice”. We propose that HPs consider dimensions of the humanisation framework as they empower those with JHS/EDS-HT to manage their own well-being.

**Table 1.** The dimensions of humanisation (Todres et al., 2009).

Humanisation—Dehumanisation Insiderness—Objectification Agency—Passivity Uniqueness—Homogenisation Togetherness—Isolation Togetherness—Isolation Sense Making—Loss of Meaning Personal Journey—Loss of Personal Journey Sense of Place—Dislocation Embodiment—Reductionism

In this next section there is a discussion relating to each dimension (Todres et al., 2009) by defining the humanising and dehumanising aspects and linking them to the experiences of those with JHS/EDS-HT. Specific case examples are employed to support the discussion, which are real cases from the authors’ practice with names changed to preserve anonymity.

## Insiderness/objectification

Insiderness and objectification occur on a continuum where insiderness is a description of the unique moods and emotions that make a person an individual. Objectification relates to a person being described or categorsied in an objective way with a label or stamp. Those with JHS/EDS-HT report being labelled as “psychosomatic” or told the symptoms they are reporting are “all in the mind” (Terry et al., 2015). This may occur when a patient presents with an acute set of symptoms that are difficult to clinically reason. The myriad of symptoms associated with JHS/EDS-HT often go beyond the label “hypermobility”.

It is suggested HPs approach those with JHS/EDS-HT in a way that recognises mood and emotions (acknowledging fear) and focuses on gaining trust and understanding (Alder, 2002; Charon, 2001; Potter, Gordon, & Hamer, 2003; Todres et al., 2009). This will enable those with JHS/EDS-HT to communicate their story and to have a voice.

James was a contemporary dance student when he attended his first appointment following the dislocation of his right patella when dancing. It had taken him three months to get an appointment and when he arrived for the consultation his knee was feeling a lot better, he was concerned about his hip pain which was preventing him from dancing. The HP explained the referral was for “knee pain” but that if the “hip pain” was to be treated another referral would be required. Situations like this have been reported and may occur because systems, processes and cultures prevent HPs considering the holistic picture of the individual. Focusing on a label, stamp or diagnosis can lead to objectification which in itself becomes a de-humanising experience. A humanising approach would involve the HP acknowledging the “complaint” and working in partnership with an individual to understand how this might be impacting on the individual’s life. This approach offers a person with JHS/EDS-HT the opportunity to communicate their concern. Working together provides an environment that supports gaining trust and mutual understanding.

## Agency/passivity

Agency is used to describe the individual choices and decisions made on a daily basis that give a sense of freedom enabling us to feel ‘human’. Passivity describes a situation in which a person finds themselves becoming an accepting recipient of something not necessarily of their choosing. This lack of choice may impact on a person’s sense of control, which contributes to feelings of anxiety. Those with JHS/EDS-HT have reported being prescribed treatments that provide little benefit and reduce their control of the situation (Terry et al., 2015), forcing them into being passive recipients of an intervention they do not understand. As HPs juggle the many competing elements of their roles there are benefits in encouraging those with JHS/EDS-HT to become active participants in the management of their condition (Alder, 2002; Charon, 2001; Peterkin, 2012).

Physiotherapists and those with JHS/EDS-HT report the benefits of continuous ongoing access to physiotherapy rather than the current referral system. It is recognised that ongoing access enables those with JHS/EDS-HT to have agency and be able to proactively address issues through self–referral, rather than responding to symptoms as a crisis. Encouraging active participation in health and well-being resulting in empowerment and a positive sense of control as an individual provides a humanised approach to managing JHS/EDS-HT.

## Uniqueness/homogenisation

Each human is an individual because of their genetic “fingerprint” and their experiences in the world, which govern perceptions of being, time and self (Todres et al., 2009). These aspects contribute to a person’s uniqueness and set them apart from their colleagues, peers or family. Homogenisation describes the way in which a group of people “fit together” and can be employed in relation to a trait or diagnosis, for example a “homogeneous” group of people with JHS/EDS-HT may be described in relation to diagnostic criteria. However, this description fails to acknowledge the uniqueness of each individual within their diagnosis in terms of symptoms and life story.

Anna suffered with “asthma” like symptoms for more than 10 years, with increasing medication. Some days she found it so hard to breathe she thought she would never be able to get enough air into her lungs, she had difficulty sleeping and was often anxious: “They say it is all in my mind,” she said “and on the days when I am struggling to breathe all I want to do is to put my head out of the car window and feel the air rush into my lungs.”

Joint Hypermobility Syndrome/Ehlers-Danlos Syndrome-Hypermobility Type is considered to be a connective tissue disorder in which there is increased extensibility of the tissues, and there maybe evidence to suggest an increased frequency of respiratory symptoms, in particular asthma. Tests indicate that alterations in the mechanical properties of the tissues may mimic and be perceived as asthmatic symptoms but they may not respond to conventional treatments (Morgan, Pearson, Davies, Gooi, & Bird, 2007).

Anna was referred to a sleep apnoea clinic and prescribed non-invasive ventilation, which enabled her to reduce her asthma medication and self-manage her symptoms. This story relates to an individual whose symptoms did not fit a homogenous diagnosis. It is suggested that a humanised approach for the management of JHS/EDS–HT requires an HP to be open to and accepting of uniqueness. The HP also needs to be able to clinically reason an individual set of symptoms in order to maximize treatment effectiveness.

## Togetherness/isolation

Togetherness and isolation occur on a continuum in our daily lives. It is normal for humans to be part of a community and to enjoy feeling a sense of belonging and togetherness. For those with JHS/EDS-HT there is anecdotal evidence that symptoms may be triggered by an event, which then has long-term consequences, eventually leading to feelings of isolation. For example symptoms may be triggered in someone who was previously asymptomatic. They may start with the dislocation of a joint, the pain associated with the event leads to a fear of movement, leading to decreased activity and a potential “domino effect” (Keer & Butler, 2010). The longer the person remains inactive, the more likely they are to become deconditioned as a result of reduced strength and endurance. Recovery may be slow with a number of setbacks (Simmonds & Keer, 2007). A continuing cycle of inactivity leads to a downward spiral of fear of pain and movement, fear of falling and of going out, a loss of confidence and an inability to leave the house (Hakim et al., 2010; Simmonds & Keer, 2008). The knock on effect of this downward spiral is like a “domino effect”, resulting eventually in increasing physical impairment (Keer & Butler, 2010), impacting on mental well-being and leading to social isolation.

The “domino effect” may also manifest in a potential loss of employment and loss of social and family roles, contributing further to isolation. Those with JHS/EDS-HT report that their ability to socialise and to get out may also be limited by fatigue. This gradual erosion of a social role may impact on a person’s identity and “sense of self”, which further impacts on well-being (Harris, Morley, & Barton, 2003). To mitigate isolation, those with JHS/EDS-HT may be encouraged to join support groups (i.e., patient-led organizations such as the Hypermobility Syndromes Association and Ehlers-Danlos Syndrome, UK (EDS-UK). In healthcare settings simple gestures like welcoming people, listening to and taking concerns seriously can make people feel less isolated (Nielsen, 2009) and provide a more humanised experience.

## Sense-making/loss of meaning

It is natural for humans to want to make sense of a situation and to try and make decisions based on that understanding. Conversely it may be confusing, frightening and frustrating if a person is unable to make sense of their situation when the diagnosis and subsequent treatment are not effective (Nielsen, 2014). It can frequently be difficult for a person to make sense of their symptoms or those of their child with JHS/EDS-HT, especially if they have heard one explanation from one HP and then a conflicting one from another (Clinch & Ecclestone, 2009).

Allowing a person to tell their story and to gain an understanding of this story is a good starting point. Those with JHS/EDS-HT often have a long history and require an opportunity to articulate their concerns and expectations. The listening skills of the HP are crucial if a consultation is to be successful. It may take the whole of the first consultation for the person to offload, tell their story and say “this is who I am” (Adler, 2002; Peterkin, 2012).

Prior to surgery Isobel wanted to make sense of what was happening in relation to her surgical procedure, and based on her previous experiences she felt she felt she needed to enable all the HPs associated with her bladder surgery to understand and make sense of her underlying condition. Isobel required surgery for bladder dysfunction, which was manifesting as urinary urgency. Bladder dysfunction is common in the general population but there may be an inherent potential for the development of urogenital problems in those with JHS/EDS-HT because of greater laxity of the pelvic floor (Norton, Baker, Sharp, & Warenski, 1995).

Isobel wanted to explain to her consultant her underlying condition JHS/EDS-HT and share with the anaesthetist her previous anaesthetic experiences and her fears. Mitigating one of these fears meant that she wanted to be able to have eye contact with the anaesthetist up to the point of sedation. This meant being allowed to wear her glasses in the operating theatre and not leave them on the ward (Knight, 2013). Attending healthcare settings can often be overwhelming and anxiety making. There may be simple processes that help a person to make sense of the situation and to share their concerns. For example in addition to providing information about an operative procedure, a humanised approach would include listening to an individual’s concerns and adapting processes accordingly.

## Personal journey/loss of personal journey

A personal journey occurs on a continuum as perceptions and experiences are compared to what has happened in the past, what is currently happening and what may happen in the future. Personal journeys are part of our life story, linking experiences and perceptions along that journey and helping us to navigate future directions. Experiences in the past and aspects of familiarity may produce feelings of comfort, while memories of discomfort or the unknown may result in feelings of anxiety and a loss of direction (Todres et al., 2009).

Those with JHS/EDS-HT have reported that diagnosis and referral to healthcare services have often been difficult and convoluted (Palmer et al., 2016). This may be because they are referred to a variety of practitioners to address “single” symptoms. Health professionals may not be open to or have understood the long-term multisystemic nature of the condition. Children, for example, may have had multiple visits to healthcare settings and experienced feelings of being doubted and in particular their perceptions of pain contested. Very often those with JHS/EDS-HT will have experienced failed interventions and in some cases an exacerbation of symptoms. In the search for an explanation or “cure” for their symptoms they may have been provided with a variety of conflicting hypotheses, some of which may or may not make sense (Clinch & Ecclestone, 2009; Palmer et al., 2016).

Continuity of care and working together with HPs are aspects that have been reported as being important for the personal journeys of those with JHS/EDS-HT. Working together requires being open to understanding that interventions may need to be adapted for individuals (Palmer et al., 2016). An example of a humanised approach requires promoting feelings of trust and comfort, providing information that enables self-management and access to care from HPs who are familiar.

## Sense of place/dislocation

Sense of place involves practices that enhance the physical environment, for example referring to surroundings that make a person “feel at home” as opposed to feeling disconnected. Physical environments that are less welcoming may make a person feel more like an outsider and “dislocated” from their surroundings.

Mary was a new mother who found herself admitted to a psychiatric hospital for post-natal depression. Health professionals were of the opinion that Mary was not coping with motherhood and her reluctance to pick up her baby was testament to this. She had explained that her wrists dislocated when she picked up her baby and that her joints had become so painful she was finding it difficult to cope with daily activities of motherhood. Health professionals perceived Mary’s behaviour to be symptomatic of post-natal depression.

Mary found herself in a physical place that didn’t make sense, a post-natal psychiatric ward where she had limited time with her newborn son and was being supervised. She had been taken away from her home situation and found herself separated from society and her family. Mary felt disconnected from the physical environment and her social and emotional situation. In this instance a humanised approach would have meant that Mary would have been able to communicate the problems her dislocating wrists were causing and her fears of dropping the baby. Health professionals would have been more open to understanding her concerns so that the real issues could have been addressed and together they would have found an effective outcome.

## Embodiment/reductionist body

Embodiment refers to aspects that enable people to expand their horizons beyond a definition of a condition and in terms of being a “passive recipient” or “diagnosis”. This concept includes enabling people to holistically achieve goals and optimise quality of life. A reductionist view of a person, for example, might focus on the known physical symptoms and not consider the broader physical, emotional and social context.

Those with JHS/EDS-HT report being disbelieved and/or dismissed because their symptoms are not understood and do not fit a pattern. Some people with JHS/EDS-HT report feeling relieved to get a diagnosis that validates their symptoms and embodies a holistic view (Palmer et al., 2016). They may have visited many different practitioners, each of whom may only have viewed one aspect in isolation:

My “journey” has highlighted how dis-jointed the approach to pelvis/back and joint problems is …. There is a desperate need for a more holistic approach where practitioners are willing [open to the idea] of secondary problems—looking at the body as a whole rather than in isolation! I live in hope! P39K (Clark, 2012, p. 163)

A humanised approach in this case is one in which HPs are open to a tangible form or description of symptoms. This requires HPs to recognise and understand the multifactorial aspects of JHS/EDS-HT and the complex central neurophysiological mechanisms that contribute to this condition (Celletti et al., 2015; Clark et al., 2014).

## Conclusion

The aim of this paper was to use the humanisation framework (Todres et al., 2009) to facilitate a discussion around the management of JHS/EDS-HT using real stories. The authors of this paper do not seek to exclude current understanding of evidence-based interventions, but seek to facilitate the delivery of these interventions in a way that adds value to the person’s experiences. In this narrative we may have blurred the boundaries of the different dimensions of the humanisation framework, each of which we have viewed along a continuum. However, the narrative provides a pragmatic approach that considers intangible aspects of those with JHS/EDS-HT within their context of place, time and emotion and in society. Those with JHS/EDS-HT report accessing many healthcare settings and may have done so throughout their lives. The nature of these settings can make people feel isolated or alone, thereby impacting on health and well-being prior to any consultation or intervention. It is important to recognize the historical challenges they may have faced in relation to their personal story, making sense of their situation in order to support the management of their condition, thus optimising well-being and quality of life.

## References

[CIT0001] Adib N., Davies R., Grahame R., Woo P., & Murray K. (2005). Joint hypermobility syndrome in childhood: A not so benign disorder? *Rheumatology*, 44(6), 744–7. doi:10.1093/rheumatology/keh557 15728418

[CIT0002] Adler H. M. (2002). The sociophysiology of caring in the doctor-patient relationship. *Journal of General Internal Medicine*, 17(11), 883–890. doi:10.1046/j.1525-1497.2002.10640.x PMC1495122 12406360

[CIT0003] Beighton P., De Pape A., Steinmann B., Tsipouras P., & Wenstrup R. J. (1998). Ehlers-Danlos Syndromes: Revised nosology, Villefranche, 1997. Ehlers-Danlos National Foundation (USA) and Ehlers-Danlos Support Group (UK). *American Journal of Medical Genetics*, 77(1), 31–37. 9557891 10.1002/(sici)1096-8628(19980428)77:1<31::aid-ajmg8>3.0.co;2-o

[CIT0004] Castori M., Morlino S., Pascolini G., Blundo C., & Grammatico P. (2015). Gastrointestinal and nutritional issues in joint hypermobility syndrome/Ehlers-Danlos syndrome-hypermobility type. *American Journal of Medical Genetics*, 169, 54–57. doi:10.1002/ajmg.c.31431 25821092

[CIT0005] Celletti C., Mari G., Ghibellini G., Celli M., Castori M., & Camerota F. (2015). Phenotypic variability in developmental coordination disorder: Clustering of generalized joint hypermobility with attention deficit/hyperactivity disorder, atypical swallowing and narrative difficulties. *American Journal of Medical Genetics Part C Seminars in Medical Genetics*, 169C, 117–122. doi:10.1002/ajmg.c.31427 25821095

[CIT0006] Charon R. (2001). Narrative medicine – a model for empathy, reflection, profession and trust. *Jama*, 286, 1897–1902. 11597295 10.1001/jama.286.15.1897

[CIT0007] Clark C. J. (2012). *Exploring the multi-factorial manifestations of joint hypermobility syndrome and the impact on quality of life*. Bournemouth, UK: Bournemouth University.

[CIT0008] Clark C. J., Khattab A. D., & Carr E. C. J. (2014). Chronic widespread pain and neurophysiological symptoms in joint hypermobility syndrome. *International Journal of Therapy and Rehabilitation*, 21(2), 60–67. doi:10.12968/ijtr.2014.21.2.60

[CIT0009] Clark C. J., & Simmonds J. V. (2011). An exploration of the prevalence of hypermobility and joint hypermobility syndrome in Omani women attending a hospital physiotherapy service. *Musculoskeletal Care*, 9, 1–10. doi:10.1002/msc.184 20645294

[CIT0010] Clinch J., & Ecclestone C. (2009). Chronic musculoskeletal pain in children: assessment and management. *Rheumatology*, 48(5), 466–474. doi:10.1093/rheumatology/kep001 19202161

[CIT0011] Connelly E., Hakim A., Davenport H. S., & Simmonds J. V. (2015). A study exploring the prevalence of joint hypermobility syndrome in patients attending a musculoskeletal triage clinic. *Physiotherapy Practice Research*, 36(1), 43–53.

[CIT0012] Farmer A., & Aziz Q. (2010). Bowel dysfunction and joint hypermobility syndrome and fibromyalgia In Hakim A., Keer R., & Grahame R. (Eds.), *Hypermobility, fibromyalgia and chronic pain*. Philadelphia, PA: Elsevier.

[CIT0013] Galvin K., & Todres L. (2013). *Caring and wellbeing: a lifeworld approach*. New York: Routledge.

[CIT0014] Grahame R. (2013). Joint hypermobility: Emerging disease or illness behaviour. *Clinical Medicine*, 13, s50–s52. doi:10.7861/clinmedicine.13-6-s50 24298184

[CIT0015] Grahame R., Bird H. A., & Child A. (2000). The revised (Brighton 1998) criteria for the diagnosis of benign joint hypermobility syndrome (BJHS). *Journal of Rheumatology*, 27, 1777–1779. 10914867

[CIT0016] Hakim A., Malfait F., De Paepe A., & Sahota A. (2010). The heritable disorders of connective tissue: Epidemiology, nosology and clinical features In Hakim A., Keer R., & Grahame R. (Eds.), *Hypermobility, fibromyalgia and chronic pain*. Philadelphia, PA: Elsevier.

[CIT0017] Harris S., Morley S., & Barton S. B. (2003). Role loss and emotional adjustment in chronic pain. *Pain*, 105, 363–370. 14499455 10.1016/s0304-3959(03)00251-3

[CIT0018] Keer R., & Butler K. (2010). Physiotherapy and occupational therapy in the hypermobile adult 143–161 In Hakim A., Keer R., & Grahame R. (Eds.), *Hypermobility, fibromyalgia and chronic pain*. Philadelphia, PA: Elsevier.

[CIT0019] Knight I. (2013). *A multidisciplinary approach to managing Ehlers-Danlos (Type III) hypermobility syndrome – Working with the chronic complex patient*. London: Singing Dragon Press.

[CIT0020] Knight I. (2015). Narrative medicine in the management of Ehlers-Danlos Syndrome – Hypermobility type. *American Journal Genetics Part C*, 169C, 123–129. doi:10.1002/ajmg.c.31428 25821096

[CIT0021] Lyell M., Simmonds J., & Deane J. (2016). A study of UK physiotherapists knowledge and training needs in hypermobility and hypermobility syndrome. *Physiotherapy Practice Research*, 37(2), 101–109. doi:10.3233/PPR-160073

[CIT0022] Malfait F., Francomano C., Byers P., Belmont J., Berglund B., Black J., … Tinkle B. (2017). The 2017 international classification of the Ehlers–Danlos syndromes. *American Journal of Medical Genetics Part C Seminars in Medical Genetics*, 175C, 8–26. doi:10.1002/ajmg.c.31552

[CIT0023] Morgan A. W., Pearson S. B., Davies S., Gooi H. C., & Bird H. (2007). Asthma and airways collapse in two heritable disorders of connective tissue. *Annals of the Rheumatic Diseases*, 66(1), 1369–1373. doi:10.1136/ard.2006.062224 17412739 PMC1994284

[CIT0024] Nielsen A. (2009). *‘It’s a Whole Lot More Than Just About My Pain’. Understanding and responding to the social dimension of living with chronic pain*. Brisbane, Australia: University of Queensland.

[CIT0025] Nielsen M. (2014). The patient’s voice In Giensven H., Strong J., & Unruh A. M. (Eds.), *Pain. A textbook for health professionalsvan*. London: Churchill Livingstone Elsevier.

[CIT0026] Norton P., Baker J., Sharp H., & Warenski J. (1995). Genitourinary prolapse and joint hypermobility in women. *Obstetric Gynecology*, 85, 225–228. doi:10.1016/0029-7844(94)00386-R 7824235

[CIT0027] Nowaczyk M. (2012). Narrative medicine in clinical genetics practice. *American Journal of Medical Genetics Part A*, 158A, 1941–1947. doi:10.1002/ajmg.a.35482 22753050

[CIT0028] Palmer S., Bailey S., Barker L., Barney L., & Elliott A. (2014). The effectiveness of therapeutic exercise for joint hypermobility syndrome: A systematic review. *Physiotherapy*, 100(3), 220–227. doi:10.1016/j.physio.2013.09.002 24238699

[CIT0029] Palmer S., Terry R., Rimes K. A., Clark C., Simmonds J., & Horwood J. (2016). Physiotherapy management of joint hypermobility syndrome – a focus group study of patient and professional perspectives. *Physiotherapy*, 102(1), 93–102. doi:10.1016/j.physio.2015.05.001 26116487

[CIT0030] Peterkin A. (2012). Practical strategies for practising narrative-based medicine. *Canadian Family Physician*, 58, 63–64. 22267625 PMC3264015

[CIT0031] Potter M., Gordon S., & Hamer P. (2003). The difficult patient in private practice physiotherapy: A qualitative study. *Journal of Physiotherapy*, 49, 53–61. 10.1016/s0004-9514(14)60188-4 12600254

[CIT0032] Simmonds J. V., & Keer R. J. (2007). Hypermobility and the hypermobility syndrome. *Manual Therapy*, 12, 298–309. doi:10.1016/j.math.2007.05.001 17643337

[CIT0033] Simmonds J. V., & Keer R. J. (2008). Hypermobility and the hypermobility syndrome, Part 2: Assessment and management of hypermobility syndrome: Illustrated via case studies. *Manual Therapy*, 13(2), e1–11. doi:10.1016/j.math.2007.11.001 18221908

[CIT0034] Terry R. H., Palmer S. T., Rimes K. A., Clark C. J., Simmonds J. V., & Horwood J. P. (2015). Living with joint hypermobility syndrome: Patient experiences of diagnosis, referral and self-care. *Family Practice*, 32(3), 354–358. doi:10.1093/fampra/cmv026 25911504 PMC4445137

[CIT0035] Tinkle B., Bird H. A., Grahame R., Lavallle M., Levy H. P., & Sillence D. (2009). The lack of clinical distinction between, the Ehlers-Danlos syndrome and the joint hypermobilty syndrome (a.k.a hypermobility syndrome). *American Journal of Medical Genetics Part A*, 149, 2368–2370. doi:10.1002/ajmg.a.33070 19842204

[CIT0036] Todres L., Galvin K., & Holloway I. (2009). The humanizationof healthcare: A value framework for qualitative research. *International Journal of Qualitative Studies on Health and Well-Being*, 4, 68–77. doi:10.1080/17482620802646204

